# Mixed fermentation improved apple juice quality: juice characteristics and mechanism

**DOI:** 10.3389/fnut.2026.1730713

**Published:** 2026-01-27

**Authors:** Keyu Lei, Yinglong Wang, Yunfeng Pu, Liling Wang, Ying Huang, Xujie Hou

**Affiliations:** 1 College of Food Science and Engineering, Tarim University, Alar, Xinjiang, China; 2 Production & Construction Group Key Laboratory of Special Agricultural Products Further Processing in Southern Xinjiang, Tarim University, Alar, Xinjiang, China

**Keywords:** apple juice, aromatic volatile compounds, fermentation, non-targeted metabolomics, overall aroma

## Abstract

**Introduction:**

Fermented apple juice demonstrates significant potential as a functional beverage with health-promoting benefits; however, optimizing its sensory quality remains challenging. This study investigated the effects of fermentation using single and mixed lactic acid bacteria (LAB) strains on the quality of ‘Red Fuji’ apple juice (AJ) from Aksu, Xinjiang.

**Methods:**

Flavor characteristics were assessed using electronic nose, electronic tongue, and HS-SPME/GC–MS, and underlying mechanisms were investigated via non-targeted metabolomics.

**Results:**

The results revealed that ‘Red Fuji’ AJ is an excellent fermentation substrate, with post-fermentation total colony counts consistently exceeding 8.5 log CFU/mL. Mixed fermentation outperformed single fermentation. At the *L. helveticus* and *L. plantarum* ratio of 1:2, yielding higher viable cell counts (>9.26 log CFU/mL), superior overall sensory acceptance (*p* < 0.05), and a more complex and pleasant flavor profile characterized by increased levels of esters, aldehydes, and alcohols, along with significantly reduced bitterness (65.3%) and astringency (74.1%). Metabolomic analysis identified key differential metabolites and metabolic pathways (amino acid metabolism, tricarboxylic acid cycle, and flavonoid biosynthesis), providing a theoretical basis for the flavor enhancement mechanism.

**Discussion:**

This study demonstrates that specific mixed LAB fermentation can effectively address common sensory acceptance issues in fermented fruit juices by simultaneously enhancing probioticviability, enriching flavor complexity, and reducing undesirable tastes.

## 1 Introduction

Apple (*Malus pumila* Mill.) belongs to the Rosaceae apple plants. Apples are rich in vitamin C, flavonoids, polyphenols, dietary fiber, organic acids, soluble sugars, pectin, minerals, and trace elements. Apples can be processed into apple juice (AJ), which can greatly retain the flavor and nutrition of apples and is becoming increasingly popular among consumers (1). As more and more consumers pursue a nutritious and healthy lifestyle, it is necessary to explore new processing technologies to enrich AJ products and improve the health benefits of apple products.

Various methods exist for the processing of AJ, including concentrated AJ, cider vinegar, and cider. Lactic acid bacteria (LAB) fermentation has received the attention of many researchers as an emerging processing method. LAB can break down nutrients from food into a variety of forms, including corresponding flavor substances, antimicrobial components, and small-molecule peptides (2). These transformations significantly alter the functional composition of the juice and enhance the bioaccessibility and bioavailability of its components (3). The associated health benefits are multifaceted and well-documented. Specific benefits include: improving and balancing the structure of intestinal flora (4); reducing insulin resistance, thereby helping prevent cardiovascular disease and type 2 diabetes (5); lowering blood glucose levels and providing hepatoprotective effects (6); and exhibiting cholesterol-lowering activity and *in vitro* bio-antagonism (7). Meanwhile, probiotic fermentation affects the aroma profile and sensory quality of fruit juices through the production and metabolism of volatile compounds such as esters, alcohols, aldehydes, ketones, and terpenoids. Probiotic fermentation has been reported to improve the organoleptic properties, especially the aroma profiles, of various fruit juices, including apple (8), pomegranate (9), peach (10), and blueberry (11), by retaining typical volatiles and producing new pleasant ones. This transformation, fundamentally a process of sugar conversion, acidification, and secondary metabolite synthesis, leads to overall improvements in the flavor, nutritional functionality, and shelf stability of juices (12).

In recent years, with the increasing demand for functional foods, research on the application of probiotics in juice processing has increased. The addition of probiotics in AJ can enhance the colour and flavor matter of AJ (8, 13). The isolation of LAB from other fermented foods and their subsequent application in apple juice fermentation not only enhances the juice’s antioxidant capacity but also promotes the generation of additional substances beneficial to human health (14, 15). The bacteriostatic effect of fermented AJ was better than the high carbohydrate, amino acid and vitamin environment suitable for the proliferation of LAB (16), and the soluble solids concentration of Aksu ‘Red Fuji’ apples is generally more than 15%, which is 1–2 percentage points higher than that of other domestic apple production areas (17), so Aksu ‘Red Fuji’ AJ is a very good LAB fermentation base. A major limitation of current research is its predominant focus on monoculture fermentation of AJ. Although mixed-culture fermentation is recognized as a more complex system that facilitates cross-feeding and metabolite exchange (10), studies on its application to AJ are scarce.

In this study, we used Aksu ‘Red Fuji’ apples as raw materials. The purpose is to develop an AJ drink in line with the concept of modern health and green, and to provide support for the industrial development of composite probiotic fermentation of AJ and the efficient use of the resources of the Aksu ‘Red Fuji’ apples.

## 2 Materials and methods

### 2.1 Materials

**Figure 1:**
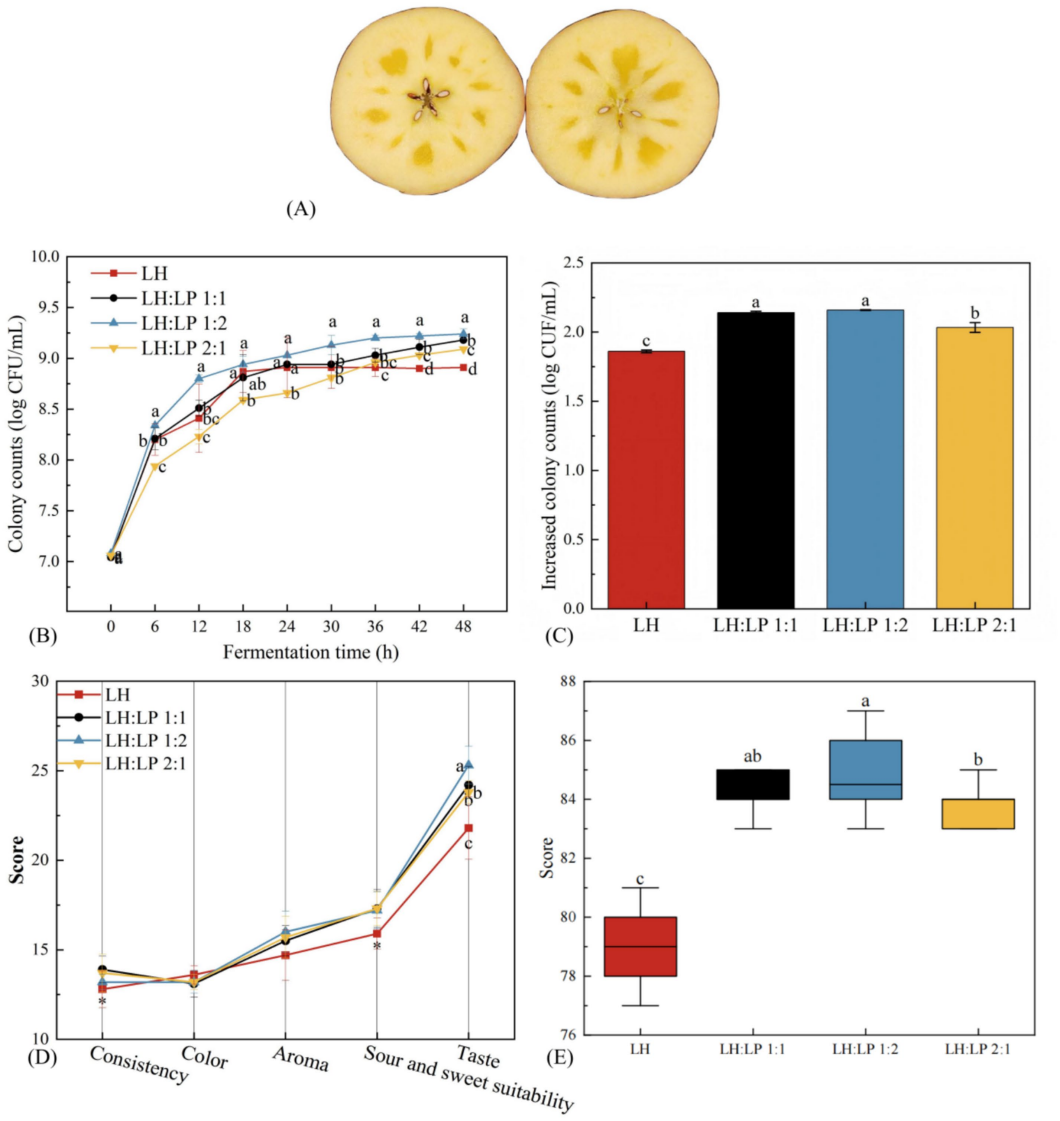
The picture of ‘Red Fuji’ apples **(A)**. Growth curves **(B)**, increased colony counts **(C)**, sensory scores **(D)**, and overall sensory scores **(E)** of fermented AJ with different composite ratio strains. Lowercase letters indicate significant differences (*p* < 0.05) among samples. * *p* < 0.05. Error bars represent the standard deviation of the mean. *n* = 3. *L. plantarum* PG-LPZ131 (LP); *L. helveticus* R0052 PG-LHR51 (LH).

LAB, including *Lactiplantibacillus plantarum* PG-LPZ131 (LP) and *Lactobacillus helveticus* R0052 PG-LHR51 (LH), were purchased from Peptide Love Biotechnology Co. (Xi’an, Shaanxi, China) as the bacterial powder. Ripe ‘Red Fuji’ apples (diameter 70–80 mm) (Figure 1A) were harvested from an apple farm in the Sixth Brigade of Alar, Xinjiang, China in October, 2024, transported to the Corps Key Laboratory of Deep Processing of Speciality Agricultural Products in Southern Xinjiang at Tarim University (Alar, Xinjiang, China) immediately and stored in 4 °C.

### 2.2 Apple juice fermentation

Apples without damage were cleaned in a fruit washer, surface dried, and manually cored. Then the apple slices were crushed using a crusher, and the crushed apple pomace was put into a juice extractor to be extracted, and the extracted apple juice was filtered with an 80 mesh strainer to obtain AJ. Finally, the filtered juice was filled into a 5.0 L fermenter, then pasteurized at 85 °C for 15 min (18) and cooled to about 40 °C before fermentation. Appropriate amounts of bacteria powder were incorporated into the sterile AJ so that the initial cell concentration in the AJ was approximately 7.0 log CFU/mL. The fermenter was kept in a water bath at 37 °C for 48 h to obtain fermented AJ. The AJ cultured under the same conditions but without the addition of LAB was used as a control check (CK). Each treatment had three replicates. Sampling was performed every 6 h by randomly collecting three 100 mL replicates from each sample. Equipment information reference Supplementary Table S1.

### 2.3 Colony counts

Determination of total colony count in AJ Reference report (19) with minor modifications. Specifically, 1.0 mL of fermented juices was diluted serially with sterile saline to 10^4^–10^5^ dilutions. Inoculation of 0.1 mL of the diluted samples was plated on the MRS Broth Agar Medium. The plates were incubated at 37 °C for 48 h. The total number of colonies was calculated using plates that exhibited colony counts between 30 and 300 CFU and showed no diffuse colony growth. The process was repeated three times, and the average value was calculated.

### 2.4 Soluble solids concentration (SSC), pH, reducing sugar, and titratable acid analysis

The SSC of AJ samples was determined using a PAL-1 Handheld Refractometer (Atago Co., Tokyo, Japan). The pH was determined using a PHSJ-3F pH meter (Shanghai Leici Co., LTD., Shanghai, China). The process was repeated three times, and the average value was calculated.

Reducing sugars (RS) in fruit juice samples were determined by direct titration. Weigh 5.0 g of the fruit juice sample, transfer it to a 250 mL volumetric flask, dilute to the mark, gently shake to ensure full mixing, prepare the test solution, and proceed with the analysis for RS. When the blue color of the solution just faded as the endpoint, the consumption volume of the sample solution was recorded. The process was repeated three times, and the average value was calculated. The unit is g·100 g^−1^.

Titratable acid (TA) was assessed using acid–base titration (20). Weigh 5.0 mL of the fruit juice sample, and transfer it to a 50 mL volumetric flask. Constant 50 mL volume, fully stirred. Upon settling, pipette 10 mL of the supernatant, 2–4 drops (10 g·L^−1^) of phenolphthalein indicator was added, and titrated with 0.01 mol·L^−1^ NaOH, expressing results as malic acid. The drop to the solution is slightly red for 30 s, it does not fade, which is the titration end point. The volume of 0.01 mol·L^−1^ NaOH solution consumed was recorded. The process was repeated three times, and the average value was calculated. The unit is g·L^−1^.

### 2.5 Sensory evaluation

This sensory evaluation procedure has been approved by the Ethics Committee for Science and Technology of Tarim University (PB20251021001). The sensory characteristics of the juice samples were evaluated according to the study of Cao (21) with slight modifications (Supplementary Table S2). Ten experts from Tarim University with experience in sensory evaluation (male to female ratio 1:1, aged between 20 and 40 years old) were organized to form a sensory evaluation panel to taste the fermented AJ. Fermented AJ was taken in 20 mL in a transparent tasting glass, randomly coded, and given to the panelists. Fermented AJ was subjected to sensory tasting in five aspects: tissue state, color, aroma, sweet and sour suitability, and taste, and the overall sensory score was the sum of the five indicators.

### 2.6 Electronic nose analysis

The odor profile of the fermented samples was analyzed using an electronic nose (C-PEN3, Beijing Enovel Technology Development Co., Ltd., Beijing, China). The experimental protocol was adapted from Ma et al. (22) with modifications. Specifically, 5.0 mL of juice was transferred into a 20 mL headspace vial and equilibrated at 25 °C for 10 min prior to analysis. The instrument’s operating parameters were set as follows: carrier gas flow rate, 400 mL·min^−1^; detection time, 80 s; and washout time, 300 s. Three sequential measurements were performed for each sample to ensure reproducibility. The sensor types of the electronic nose and their corresponding response components are provided in Supplementary Table S4.

### 2.7 Electronic tongue analysis

The taste profile of the fermented samples was analyzed using an electronic tongue (SA402B, Intelligent Sensor Technology, Inc., Atsugi, Japan). The experimental method was adapted from Lv et al. (23). Food five flavor sensors C00, AE1, CA0, CT0, AAE test four times, remove the first cycle to take the average value of the last three times as the test results. The sensor-to-taste value mapping information is shown in Supplementary Table S5.

### 2.8 Volatile organic compound (VOC) analysis

Headspace-solid phase microextraction gas chromatography–mass spectrometry (HS-SPME/GC–MS) was used for the qualitative and quantitative analysis of VOCs in fruit juice samples. The method was slightly modified with reference to that of Gao et al. (16). The detected mass spectral data were used for the preliminary identification of VOCs by comparing with the mass spectra in the standard NIST 17 library. VOCs with a match of 70% or more were selected for analysis. The volatile components of the samples were quantitatively analyzed by the internal standard method using cyclohexanone as the internal standard, and the relative concentrations of the volatile components were calculated by the formula based on the concentrations of the standards and the peak areas. Three replicates were performed for each sample. All biological replicates per sample were analyzed in triplicate. A balanced mixture of all sample extractions (including three replicates) was settled as a mixed sample for quality control (QC).

### 2.9 Non-targeted metabolomics analysis

The samples were sent to Shanghai Paisenuo Biotechnology Co., Ltd. for analysis. The testing method referenced Want et al. (24), as detailed below: Transfer 600 μL of the sample to a 1.5 mL centrifuge tube and centrifuge at 16,700 × g at 4 °C for 15 min. Transfer 400 μL of the supernatant to a 2 mL centrifuge tube, freeze it rapidly in liquid nitrogen, and then freeze-dry it using a freeze-dryer. Add 1.0 mL of pre-chilled methanol: acetonitrile (1:1, v/v), vortex for 30 s. Freeze at −20 °C for 30 min. Centrifuge at 16,700 × g and 4 °C for 10 min, then vacuum concentrate 850 μL of the supernatant to dryness. Add 150 μL of 50% methanol (containing 5 ppm 2-chlorophenylalanine) to resuspend, vortex for 30 s. Centrifuge at 16,700 × g and 4 °C for 10 min, transfer the supernatant through a 0.22 μm filter membrane, and add the filtrate to the detection vial. Mix 10–20 μL of each sample filtrate to form a QC sample for evaluating instrument stability and data reliability.

Use an ACQUITY UPLC HSS T3 column (100 Å, 1.8 μm, 2.1 mm × 100 mm) with a flow rate of 0.4 mL/min, column temperature of 40 °C, autosampler temperature of 8 °C, and injection volume of 2.0 μL. Mobile phase for both positive and negative modes: Mobile phase A is 0.1% formic acid in water, and mobile phase B is acetonitrile (containing 0.1% formic acid). The elution gradient is as shown in Supplementary Table S3.

The Thermo Orbitrap Exploris 120 mass spectrometer was used to collect DDA mass spectrometry data in both positive and negative ion modes under the control of the Xcalibur software (version 4.7, Thermo). Using an HESI source, spray voltage 3.5 kV/−3.0 kV, sheath gas 40 arb, auxiliary gas 10 arb, capillary temperature 320 °C, auxiliary gas temperature 300 °C, primary resolution 60,000, scan range 70–1,000 m/z, AGC Target Standard, Max IT 100 ms, selecting the top 4 ions for secondary fragmentation, dynamic exclusion time 4 s, secondary resolution 15,000, HCD collision energy 30%, AGC Target Standard, Max IT Auto. All formal samples and QC samples are analyzed using the aforementioned chromatographic and mass spectrometric methods. Prior to formal sample injection, 2–4 QC samples are injected to balance the system. During sample injection, one QC sample is injected every 5–10 samples. For 5 samples or fewer, QC is not performed, and the data is used for subsequent data evaluation and quality control.

Import the raw format data from the instrument into the MS-DIAL software (version 4.9.221218) and perform peak extraction, alignment, filtering, and metabolite identification using this software. Peaks not detected in QC samples with a QC rate greater than 50% were filtered out. The undetected peaks were filled with missing values using the software’s Gap Filling algorithm and then normalized. Metabolite identification was based on the PerSonalbio Next-Generation Metabolomics Database (PSNGM), which includes a custom-built standard library, the mzCloud database,^1^ LIPIDMAPS,^2^ HMDB,^3^ MoNA,^4^ NIST_2020_MSMS, and AI-predicted MSMS spectral libraries.

### 2.10 Statistical analysis

Data were systematically organized using Excel 2019. SPSS 27.0.1 (IBM Corporation, Armonk, NY, United States) was used for ANOVA, multiple comparisons, and independent samples *t*-test. Data are expressed as mean ± standard deviation (SD). Data visualization was conducted with Origin 2021 software (OriginLab Corporation, Northampton, MA, United States) and TBtools-II (48). Metabolomics data analysis and graphing were performed using the Paisenuo Genescloud,^5^ an online platform for data analysis.

## 3 Results and discussion

### 3.1 Bacterial growth

Previous experiments by the research team have demonstrated that LH is an excellent strain for AJ, effectively enhancing its overall sensory score. AJ serves as an excellent medium for LP. Existing research has confirmed that LP and LH can coexist effectively and undergo fermentation together (25). The LH and the LP were inoculated in the ratios of 2:1, 1:1, and 1:2 to ensure that the initial bacterial concentrations in the juice were all 7 log CFU/mL, and the juice was fermented at 37 °C for 48 h. All samples met the min survival level of probiotics that can have a beneficial effect on host health after passing through the digestive tract (2). Figures 1B,C show that the total colony count in AJ depended on the inoculation ratio. Notably, the LH + LP co-fermentation resulted in a higher colony count compared to the monoculture fermentation with LH only. When LH: LP was 1:2, the colony count reached the maximum value of 9.26 log CFU/mL at the end of fermentation, which was 30.5% higher than the pre-fermentation value, and this increase was higher than that of 26.4% in the LH monoorganism fermentation.

### 3.2 Sensory scores

The sensory evaluation of mixed fermented AJ with different ratios was performed according to the sensory evaluation table, as shown in Figures 1D,E. The scores of mixed fermentation were higher than those of single fermentation (*p* < 0.05), which indicated that the mixed fermentation could improve the sensory qualities of AJ. The differences were not significant between LH:LP = 1:2 and LH:LP = 1:1, and the differences were significant between LH:LP = 1:2 and LH:LP = 2:1 (*p* < 0.05), and the differences between LH:LP = 1:1 and LH:LP = 2:1 were significant (*p* < 0.05). However, LH:LP of 1:2 gave better scores for aroma and flavor, and better fermentation characteristics for this ratio. Consequently, the LH:LP = 1:2 ratio, identified as optimal, was adopted for all subsequent studies. The 2:1 and 1:1 ratios were not investigated further.

Based on the above results, LHAJ was obtained by single fermentation with LH, and HPAJ was obtained by mixed fermentation with LH:LP = 1:2. Taking the CK as the control, the flavor and taste characteristics of LHAJ and HPAJ were compared, and to further elucidate the effect of fermentation on AJ by qualitatively quantifying the volatiles.

### 3.3 SSC, pH, reducing sugars (RS), and titratable acids (TA) results

**Figure 2:**
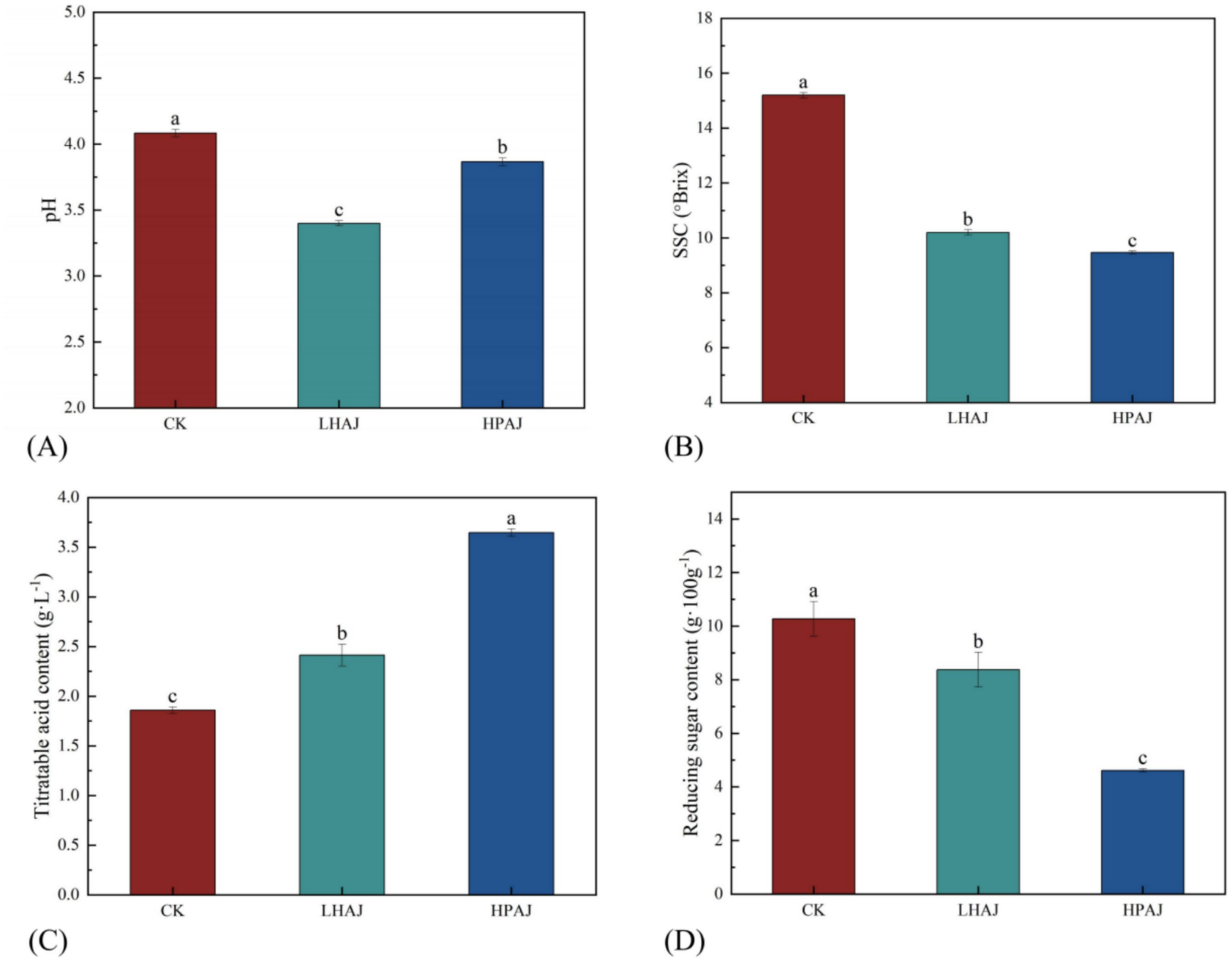
pH **(A)**, SSC **(B)**, reducing sugar **(C)**, and titratable acid **(D)** of AJ samples. Lowercase letters indicate significant differences (*p* < 0.05) among AJ samples. Error bars represent the standard deviation of the mean. *n* = 3. *L. helveticus* R0052 fermented AJ (LHAJ); *L. helveticus* R0052 and *L. plantarum* fermented AJ as 1:2 (HPAJ).

As shown in Figure 2, the pH, SSC, RS, and TA of all fermentations were significantly changed (*p* < 0.05) compared to CK, which indicates that LAB fermentation caused the depletion of carbohydrates and the production of acids in the juice matrix (11, 20, 26). After fermentation, both LHAJ and HPAJ significantly reduced SSC. LHAJ exhibited a greater pH reduction, while HPAJ showed a smaller decrease. The results indicate that HPAJ exhibits significantly lower RS than LHAJ, showing a 36.6% decrease compared to CK. HPAJ contains higher TA than LHAJ, exceeding CK by 66.5%. Mixed fermentation demonstrates superior efficiency in consuming sugars present in AJ. This increased acid production, in turn, led to a decrease in both SSC and pH. Notably, despite having higher TA, HPAJ maintains a lower pH than LHAJ. This suggests that HPAJ produces a distinct acid profile, likely characterized by a greater proportion of stronger organic acids or a different balance of acids and their buffering salts.

### 3.4 Volatility analysis

#### 3.4.1 Electronic nose analysis results

**Figure 3:**
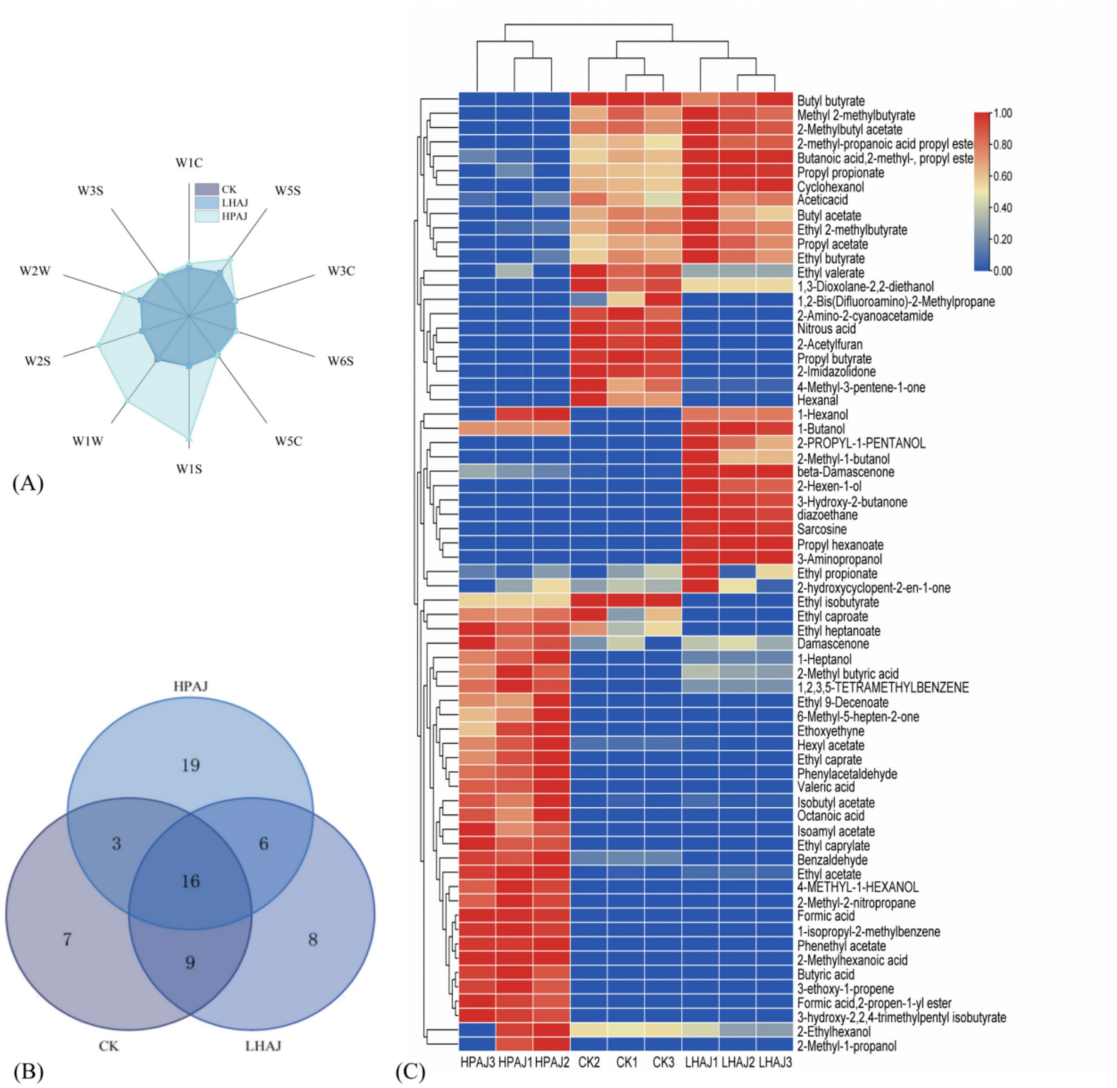
Radar chart for flavor profile analysis **(A)**, Venn diagram of flavor substances **(B)**, and heat map analysis of different fermented AJ samples **(C)**. *n* = 3. In the heat map, a gradient from blue to red indicates an increase in the standardized value from low to high. *L. helveticus* R0052 fermented AJ (LHAJ); *L. helveticus* R0052 and *L. plantarum* fermented AJ as 1:2 (HPAJ).

The aroma of a product can significantly influence consumer purchasing decisions. Numerous studies have demonstrated that LAB fermentation enriches the volatile components in fruit juices and enhances their aromatic profiles. As illustrated in Figure 3A and Supplementary Table S4, the sensors W5S (high sensitivity, particularly responsive to nitrogen oxides), W1S (sensitive to methyl compounds), W1W (responsive to sulfur compounds), W2S (sensitive to alcohols, aldehydes, and ketones), and W2W (aromatic components, reactive to organic sulfides) exhibited better performance. Notably, HPAJ exhibited heightened responses in the W1S, W1W, and W2S sensors. These sensors are typically associated with methyl compounds, which contribute fruity and sweet notes, thereby enriching the aromatic profile. Simultaneously, trace sulfur compounds enhance tropical nuances, while alcohols balance fermented tones with floral hints. The presence of aldehydes and ketones further shapes the aroma spectrum, driving it toward either fresh or mature fruity characters. Consequently, HPAJ fermentation effectively improves the overall aroma complexity of apple juice.

#### 3.4.2 Volatile components results

LAB fermentation can add many flavorful substances to AJ and improve the original flavor (27), and can produce many acids, lipids, and alcohols. As shown in Figure 3B, 35, 39, and 44 aroma substances were detected in the three products, 8 and 19 aroma substances unique to LHAJ and HPAJ, respectively. There were differences in the aroma concentrations of esters, alcohols, and aldehydes between the different fermentations.

Esters are the most widespread and important compounds present in AJ and are responsible for giving fermented AJ a richer taste, aroma, and flavor. As shown in Table 1, HPAJ contains 21 kinds of esters, LHAJ contains 18 kinds of esters, and CK contains 21 kinds of esters. Esters, as the main volatile components in AJ, make AJ have pleasant odors such as floral, fruity, ester, and sweet aromas. The concentrations of propyl acetate, isobutyl acetate, ethyl caprylate, and ethyl caprate increased significantly after fermentation of AJ (*p* < 0.05), while mixed fermentation produced more hexyl acetate, isoamyl acetate, and ethyl caprylate, etc. (*p* < 0.05), among which ethyl caprylate, phenyl ethyl acetate, and isoamyl acetate were higher in the composite fermentation products, and they mainly represented fruity, aniseed, floral, banana, and pear odors. Hexyl acetate is used in the formulation of apple flavors due to its high aroma and is often used in the formulation of apple flavors. It has been shown that methyl benzoate, butyl acetate, and hexyl acetate are the main esters in fermented AJ (28), and the concentration of hexyl acetate in HPAJ was much higher than that in CK (higher by 81.8%, *p* < 0.05), which indicates that mixed fermentation can promote the production of the main flavoring substances in AJ, and enhance the richness of the aroma of AJ.

**Table 1:** Aromatic volatile compounds in CK, LHAJ, and HPAJ.

	Volatile compounds	Retention time (min)	Concentrations (μg·L^−1^)		
			CK	LHAJ	HPAJ
Esters					
1	Ethyl acetate	7.15	112 ± 14^c^	169 ± 7^b^	1,030 ± 23^a^
2	Formic acid, 2-propen-1-yl ester	7.76	ND	ND	1728 ± 145
3	Ethyl propionate	8.50	163 ± 17^a^	189 ± 40^a^	156 ± 11^a^
4	Propyl acetate	8.89	200 ± 18^b^	276 ± 40^a^	ND
5	Methyl 2-methylbutyrate	9.84	47.8 ± 6.8^b^	58.6 ± 5.2^a^	ND
6	Isobutyl acetate	9.85	33.6 ± 5.0^b^	38.4 ± 6.4^b^	207 ± 30^a^
7	Ethyl butyrate	10.65	775 ± 63^a^	901 ± 98^a^	344 ± 53^b^
8	Ethyl isobutyrate	10.68	133 ± 0^a^	ND	74.03 ± 0.02^b^
9	Propyl propionate	10.79	100 ± 6^b^	163 ± 3^a^	28.1 ± 0.9^c^
10	Ethyl 2-methylbutyrate	11.10	1850 ± 151^a^	2070 ± 238^a^	526 ± 114^b^
11	Butyl acetate	11.87	1,180 ± 145^a^	1,240 ± 360^a^	ND
12	2-Methylbutyl acetate	13.40	3,350 ± 233^b^	3,990 ± 237^a^	ND
13	Isoamyl acetate	13.42	ND	ND	6,810 ± 1,010
14	Propyl butyrate	13.45	109 ± 4	ND	ND
15	Ethyl valerate	13.69	123 ± 13^a^	37 ± 2^b^	42.6 ± 4.6^b^
16	Butanoic acid,2-methyl-, propyl ester	14.02	453 ± 25^b^	632 ± 2^a^	195 ± 38^c^
17	Acetic acid	15.08	75.1 ± 15.3^a^	93 ± 10^a^	28.9 ± 9.3^b^
18	Butyl butyrate	16.77	80.8 ± 1.6^a^	72.1 ± 12.8^a^	ND
19	2-Methyl-propanoic acid propyl ester	16.82	260 ± 25^b^	408 ± 34^a^	ND
20	Ethyl caproate	17.48	6,430 ± 2750^a^	1950 ± 13^b^	7,540 ± 284^a^
21	Hexyl acetate	18.90	580 ± 7.76^b^	317 ± 39^b^	3,190 ± 400^a^
22	Propyl hexanoate	20.62	ND	49.3 ± 0.4	ND
23	Nitrous acid	20.64	834 ± 42	ND	ND
24	Formic acid	20.72	ND	ND	3,700 ± 80
25	Ethyl heptanoate	21.21	113 ± 57^b^	ND	196 ± 18^a^
26	Ethyl caprylate	25.41	ND	ND	17,300 ± 1,210
27	Ethyl caprate	36.69	ND	ND	632 ± 95.1
28	Ethyl 9-decenoate	38.84	ND	ND	258 ± 51
29	Phenethyl acetate	43.01	ND	ND	8,050 ± 147
30	3-Hydroxy-2,2,4-trimethylpentyl isobutyrate	44.27	ND	ND	32.1 ± 2.1
Alcohol					
31	3-Aminopropanol	10.79	ND	162 ± 1	ND
32	2-Methyl-1-propanol	12.17	ND	ND	337 ± 24.7
33	1-Butanol	13.88	ND	737 ± 22^a^	552 ± 2^b^
34	2-Methyl-1-butanol	16.16	ND	910 ± 265	ND
35	3-Methyl-1-butanol	17.76	ND	51.9 ± 15.3	ND
36	1-Hexanol	21.72	ND	4,310 ± 130^b^	5,290 ± 208^a^
37	Cyclohexanol	23.85	410 ± 24^b^	651 ± 10^a^	ND
38	2-Hexen-1-ol	23.85	ND	591 ± 49	ND
39	1,3-Dioxolane-2,2-diethanol	24.37	65.6 ± 8.4^a^	38.7 ± 0.0^b^	ND
40	1-Heptanol	26.02	ND	31.9 ± 0.2^b^	188 ± 25^a^
41	4-Methyl-1-hexanol	26.03	ND	ND	168 ± 17
42	2-Propyl-1-pentanol	27.81	ND	ND	431 ± 32
43	2-Ethylhexanol	27.82	359 ± 18^b^	214 ± 75^c^	676 ± 24^a^
Ketones					
44	3-Hydroxy-2-butanone	17.44	ND	1880 ± 98	ND
45	2-Hydroxycyclopent-2-en-1-one	19.93	3,730 ± 90^a^	3,930 ± 651^a^	3,690 ± 366^a^
46	6-Methyl-5-hepten-2-one	21.42	ND	ND	154 ± 38
47	2-Imidazolidone	36.60	55.2 ± 3.1	ND	ND
48	beta-Damascenone	43.17	223 ± 3^c^	386 ± 0^a^	256 ± 16^b^
49	Damascenone	43.18	205 ± 23^b^	218 ± 9^b^	260 ± 10^a^
Aldehydes					
50	Hexanal	11.99	471 ± 93	ND	ND
51	4-Methyl-3-pentene-1-one	16.97	2,160 ± 590^a^	124 ± 2^b^	ND
52	Benzaldehyde	30.61	76.6 ± 7.5^b^	ND	479 ± 37^a^
53	Phenylacetaldehyde	37.15	ND	ND	9,950 ± 1,020
Acids					
54	Sarcosine	13.30	ND	184 ± 4	ND
55	2-Methylhexanoic acid	38.20	198 ± 3^c^	203 ± 3^b^	952 ± 14^a^
56	2-Methyl butyric acid	38.21	ND	200 ± 40^b^	607 ± 126^a^
57	Valeric acid	43.76	ND	ND	610 ± 47
58	Butyric acid	43.77	ND	ND	368 ± 30
59	Octanoic acid	48.48	ND	ND	971 ± 145
Others					
60	3-Ethoxy-1-propene	7.76	ND	ND	279 ± 24
61	1,2-Bis(difluoroamino)-2-methylpropane	11.76	362 ± 386	ND	ND
62	2-Amino-2-cyanoacetamide	13.86	649 ± 72	ND	ND
63	Diazoethane	13.90	ND	1,026 ± 45	ND
64	2-Methyl-2-nitropropane	16.34	ND	ND	9,980 ± 1,047
65	Ethoxyethyne	16.38	ND	87.4 ± 4.7^b^	31,100 ± 7970^a^
66	1-Isopropyl-2-methylbenzene	25.80	ND	ND	454 ± 16
67	1,2,3,5-Tetramethylbenzene	25.84	ND	95.5 ± 2.8^b^	425 ± 57^a^
68	2-Acetylfuran	26.37	28.2 ± 1.0	ND	ND

Results are expressed as mean ± standard deviation, *n* = 3. ND, not detected. Different letters in the same row signify significant differences (*p* < 0.05). *L. helveticus* R0052 fermented AJ (LHAJ); *L. helveticus* R0052 and *L. plantarum* fermented AJ as 1:2 (HPAJ).

Microorganisms are able to produce many alcohols through sugar metabolism, dehydrodecarboxylation of amino acids, etc. Alcohols are also precursors of ester compounds (29–31). A moderate amount of alcohol can make AJ have a unique aroma. It has been pointed out that glucose and fructose metabolism through glycolysis and the acetaldehyde pathway are able to produce ethanol, which imparts enzyme wine aroma (32). As can be seen in Table 1, a total of 14 alcohols were detected in the three products, including 3 alcohols in CK, 10 in LHAJ, and 7 in HPAJ. In terms of quantity, AJ fermented by LAB was able to produce more alcohols, whereas the number of alcohols decreased and changed in type after mixed fermentation with multiple bacteria. 1-Hexanol (*p* < 0.05) with strong grassy, fruity, and alcoholic odor, 2-ethylhexanol (*p* < 0.05) with a sweet fatty aroma and soft floral alcoholic note, and 2-methyl-1-propanol (*p* < 0.05) with an alcoholic taste and a grassy flavor were the main alcohols in HPAJ (33), and the highest concentration was n-hexanol at 5,292 μg·L^−1^, which make HPAJ have unique aroma.

In addition to these higher concentrations, some lower alcohols, such as 4-methyl-1-hexanol and heptanol, are also present in HPAJ. It has been shown that the lower alcohols, although not in high concentration in fermented AJ, have a low aroma threshold and are important volatile components of fermented AJ, whereas too high a concentration also produces undesirable odors (34).

The carbonyls in enzymes are dominated by ketones and aldehydes, most of which are produced by bacteria. Most aldehydes are synthesized through amino acid metabolism and lipid oxidation (35). Excess aldehydes may cause off-flavors, but when organically combined with other oxides, they are able to enhance the aroma of enzymes. In Table 1, it can be seen that after single fermentation, fermented AJ produced more 3-hydroxy-2-butanone with cheese aroma, and after mixed fermentation, there was a high level of phenylacetaldehyde in fermented AJ, which makes fermented AJ have the aroma of hyacinth and lilac, an odor that is not contained in AJ.

Acids are by-products of LAB metabolism and are not only important aroma and flavor-presenting substances in AJ, but also important precursors of ester compounds, which can be used to flavor apple, pineapple, pear, strawberry, and other fruit flavors. A moderate amount of acid can impart fruit and vegetable cheese flavors, but too much acid can lead to deterioration of flavor during fermentation, thus reducing product mouthfeel. As shown in Table 1, the number and concentration of acids in AJ increased significantly after fermentation. In addition to the original 2-methylhexanoic acid, which increased by 79.2% (*p* < 0.05), octanoic acid and 2-methyl butyric acid (*p* < 0.05) with fruity aroma increased more in HPAJ to 971 μg·L^−1^ and 633 μg·L^−1^, respectively. In addition to the positive odor, valeric acid with a rotting sour aroma was also produced. Butyric acid is a major member of the short-chain fatty acids, and butyrate ions, as well as similar butyric acid ions, may not only enhance human and intestinal health in many other animal species, but also dehydrate with alcohols to generate esters with pleasant fruit flavors and esters (36).

Taken together, the kinds of volatiles and aroma kinds of HPAJ are more abundant (Figure 3; Table 1). Alcohols and esters were the main flavor components. The kinds of aldehydes, ketones, and acids, although less, the presence of these substances also brought more aroma components to AJ (35), which enhanced the richness and three-dimensionality of AJ aroma, and their interactions produced a harmonious aroma, which had a positive impact on the flavor of the product. In addition to common aroma components such as esters, alcohols, aldehydes and ketones, and acids, AJ also produces some other classes of volatile substances, ethers, alkanes, and so on.

### 3.5 Non-volatile analysis

#### 3.5.1 Electronic tongue analysis results

**Figure 4:**
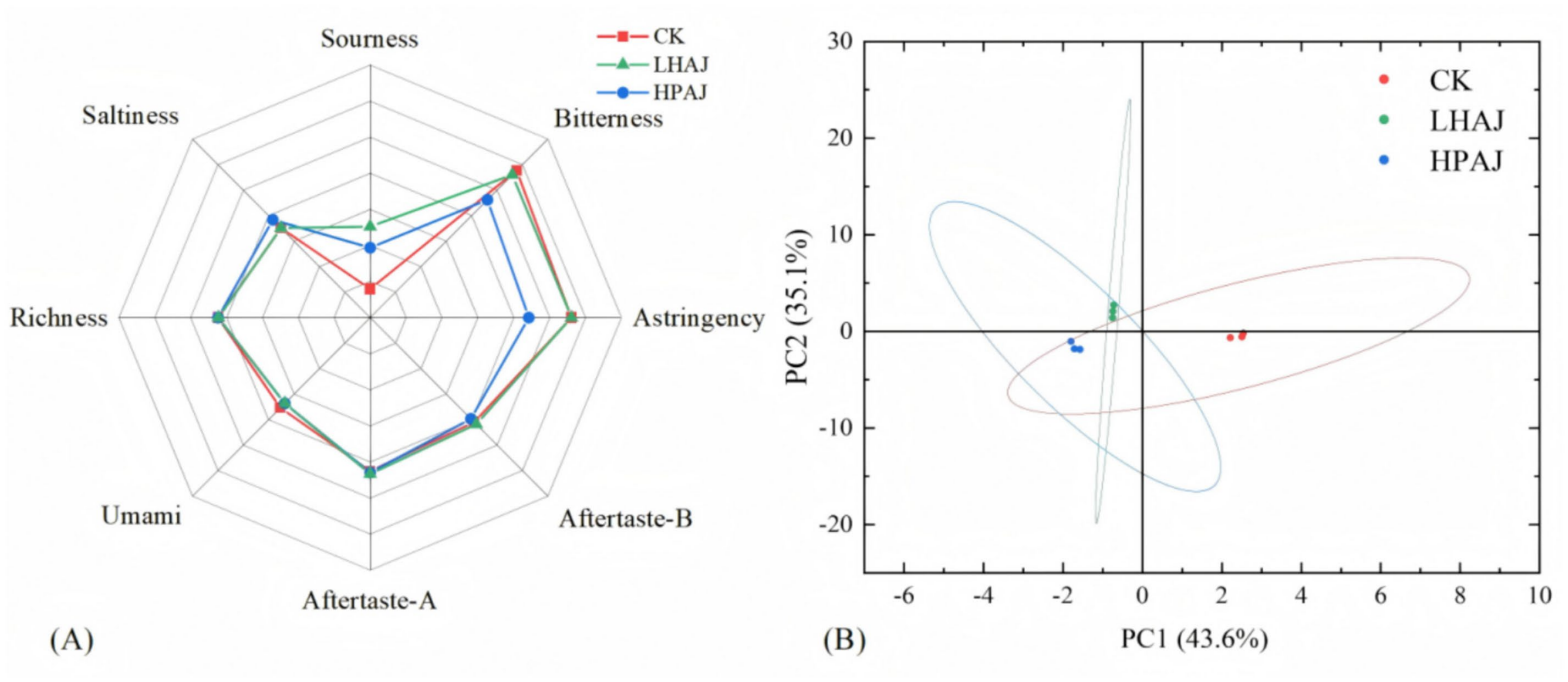
Radar map of flavor analysis **(A)** and PCA analysis **(B)** for different fermented AJ samples. *L. helveticus* R0052 fermented AJ (LHAJ); *L. helveticus* R0052 and *L. plantarum* fermented AJ as 1:2 (HPAJ).

An electronic tongue was used to assess the differences in flavor characteristics of fermented AJs. The electronic tongue was used to analyze the sensory attributes of the samples by means of taste sensors, and the taste radargrams of different fermented AJs were established by extracting the response values of each sensor. As shown in Figure 4A, sourness: LHAJ > HPAJ > CK; bitterness: CK > LHAJ > HPAJ; astringency: LHAJ > CK > HPAJ. The bitter and astringent flavors of HPAJ were lower than those of CK and LHAJ, indicating that the mixed fermentation reduced the bitterness (65.3%) and astringency (74.1%) of CK, but LHAJ only reduced the bitterness (9.6%). Bitter aftertaste reflected the degree of residual bitterness, with HPAJ not having a bitter aftertaste, and LHAJ having the greatest, with a 75.0% increase from CK. Sourness increased by 54.0% after single fermentation and 35.6% after mixed fermentation. Meanwhile, bitterness was reduced more after mixed fermentation than after single fermentation by 65.3%. The measurements of savory flavor were all less than the point of no flavor, which means that all three samples had no savory flavor, so no comparison was made. There were no differences in the other taste indicators. LAB fermentation effectively reduced the bitterness and astringency of CK, leading to a significant improvement in its overall taste. As concluded in Section 3.3, LHAJ exhibits a lower pH than HPAJ, which typically translates to a more pronounced and pungent acidity in LHAJ, whereas HPAJ delivers a comparatively milder sourness. This result is well validated by the results from the electronic tongue.

Further PCA analyses were performed on different AJ products. As shown in Figure 4B, the contribution of PC_1_ of the sample was 43.6% and that of PC_2_ was 35.1%, and the total contribution of the two PCs was greater than 75.0%, indicating that the electronic tongue can well reflect the variability among different AJ products. In Figure 4B, CK is located in the fourth quadrant, HPAJ is located in the third quadrant, and LHAJ is located in the second quadrant. It can be seen that the electronic tongue test with PC analysis can distinguish each AJ product well and reflect the characteristics of the samples well with differences.

#### 3.5.2 Differential accumulation metabolite analysis

To obtain differences accumulate metabolites (DAMs) between different juice samples, OPLS-DA analysis was performed (Supplementary Figure S1), with Q_2_ (representing predictive ability) > 0.8, indicating that the model demonstrated good performance and reliable results. Further pairwise comparisons were conducted on the juice samples. In the comparison between CK and LHAJ, a total of 550 DAMs were identified, including 339 significantly upregulated [255 in the positive ion mode (POS) and 84 in the negative ion mode (NEG)] and 211 significantly downregulated (132 in POS and 79 in NEG). In the comparison between CK and HPAJ, a total of 264 DAMs were identified, including 93 significantly upregulated (67 in POS and 26 in NEG) and 171 significantly downregulated (89 in POS and 82 in NEG). In the comparison between LHAJ and HPAJ, a total of 389 DAMs were identified, including 112 significantly upregulated (77 in POS and 35 in NEG) and 277 significantly downregulated (190 in POS and 87 in NEG).

**Figure 5:**
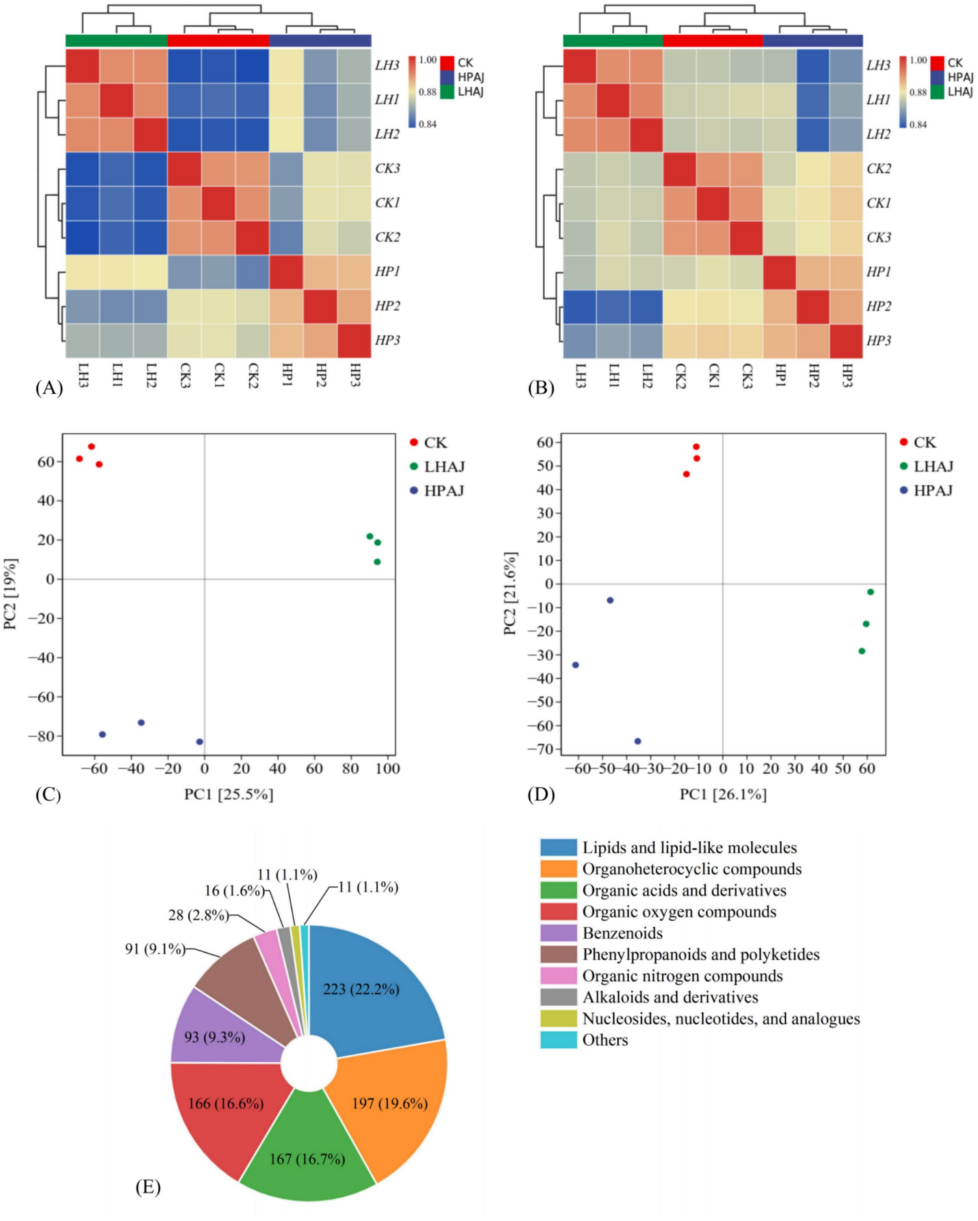
Sample correlation heat map [POS **(A)**, NEG **(B)**]. Sample PCA score chart [POS **(C)**; NEG **(D)**]. Compound classification statistical diagram **(E)**. *n* = 3. *L. helveticus* R00 52 fermented AJ (LHAJ). *L. helveticus* R0052 and *L. plantarum* fermented AJ as 1:2 (HPAJ).

Further analysis of the differences in metabolites between different groups of fermented AJ, as shown in Figures 5A,B, revealed that samples within the same group exhibited high similarity and good correlation, while significant differences were observed between groups. Additionally, PCA analysis also indicated differences between groups (Figures 5C,D), with the PC_1_ accounting for 25.5% (POS) and 26.1% (NEG), and the PC_2_ accounting for 19.0% (POS) and 21.6% (NEG). In terms of overall metabolites, HPAJ annotated more metabolites than CK and LHAJ. In the electronic nose analysis, HPAJ occupied the largest area in the radar chart, indicating that it contains more flavor compounds or higher concentrations of them, which is consistent with the metabolomics results.

#### 3.5.3 Metabolite information annotation

As shown in Figure 5E, through database annotation of metabolites, a total of 1,003 metabolites were identified in all AJ samples, including 223 lipid and lipoid molecules (22.2%), 197 organic heterocyclic compounds (19.6%), 167 organic acids and their derivatives (16.7%), 166 organic oxygen compounds (16.5%), 93 benzene compounds (9.3%), 91 phenylacetone and polyketone compounds (9.1%), 28 organic nitrogen compounds (2.8%), 16 alkaloids and their derivatives (1.6%), 11 nucleosides, nucleotides, and their analogues (1.1%), and 11 other types of substances (1.1%). GC–MS results also indicated that esters were the most abundant, consistent with the predominance of lipid and lipoid molecules in metabolomics. The alcohols, ketones, and aldehydes identified in the GC–MS results correspond to organic oxides in metabolomics, while the acidic compounds are similar to organic acids and their derivatives in metabolomics. The nitrogen oxides detected by the electronic nose correspond to organic heterocyclic compounds, benzene compounds, phenylpropanoids, and polyketides in metabolomics.

**Figure 6:**
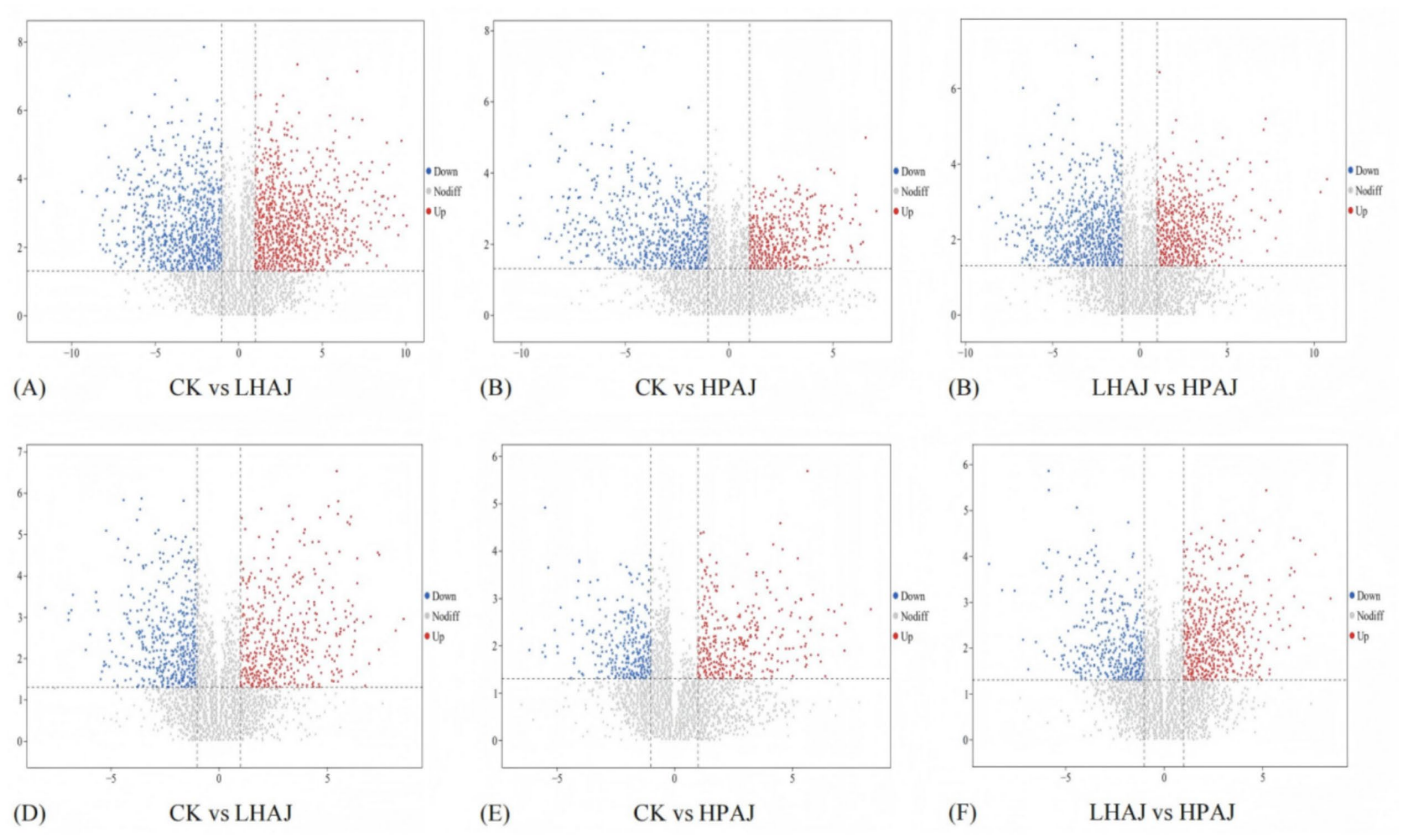
CK vs. LHAJ, CK vs. HPAJ, LHAJ vs. HPAJ differential metabolite volcano map [POS **(A–C)**; NEG **(D–F)**]. *n* = 3. *L. helveticus* R0052 fermented AJ (LHAJ). *L. helveticus* R0052 and *L. plantarum* fermented AJ as 1:2 (HPAJ).

Volcano plots can visually display metabolites that show significant changes under different fermentation conditions, aiding researchers in identifying and screening metabolites with biological significance. Based on t-tests with *p*-values <0.05 and OPLS-DA models with VIP scores ≥1, different metabolites were identified, resulting in the volcano plot shown in Figure 6. As shown in Figure 6, under single fermentation conditions, there were more upregulated metabolites than downregulated metabolites. Notably, compared to mixed fermentation conditions, mixed fermentation conditions had more upregulated metabolites. Specifically, compared to LHAJ, the most significantly different substances among the up-regulated substances in POS were Cribrarione B and Echiguanine B. Cribrarione B is a novel naphthoquinone pigment (37), and Echiguanine B is an alkaloid. Under NEG conditions, the most significantly different up-regulated substances were (2E)-2-Isopropyl-2-butenedioic acid, an unsaturated dicarboxylic acid derivative with potential as a multi-target bioactive molecule, and Succinic acid, a key intermediate metabolite in the tricarboxylic acid cycle (TCA cycle). The most significantly different substance among the downregulated substances was 4-[5-[[4-[5-[acetyl(hydroxy)amino]pentylamino]-4-oxobutanoyl]-hydroxyamino]pentylamino]-4-oxobutanoic acid. This substance is an iron chelator that can remove free iron and inhibit ROS, but excessive chelation of serum iron (Fe^3+^) can block hemoglobin synthesis, leading to the accumulation of the heme precursor *δ*-amino-*γ*-ketopentanoic acid (ALA) and resulting in iron-deficiency anemia (IDA). The substance with the largest downregulation fold change (blue point farthest to the left) is epicatechin, which is a phenolic compound with antioxidant activity, exhibiting potent antioxidant, anti-inflammatory, and anticancer activities (38). It is a heat-sensitive flavonoid (39). Additionally, epicatechin was significantly upregulated in the comparison between LHAJ and HPAJ, indicating that more epicatechin is retained in HPAJ. The substance with the largest upregulation difference (red point on the far right) in NEG is ethyl lactate, which is widely present in fermented foods. It can scavenge free radicals and inhibit inflammatory factors, and it has a unique aroma of rum, fruit, and cream. This substance also showed significant upregulation in GC–MS measurement results. In the comparison between CK and HPAJ, under POS conditions, the most significantly downregulated substance in CK is L-isoleucyl-L-threonine, which is an amino acid derivative that inhibits the production of pro-inflammatory factors such as TNF-*α* and IL-6 by blocking TLR4 receptor dimerization and inhibiting NF-κB nuclear translocation. It also enhances IL-10 secretion and suppresses excessive Th1 immune responses, thereby alleviating autoimmune colitis. Additionally, the content of L-isoleucyl-L-threonine was downregulated under fermentation conditions. Among the downregulated substances, the most significant was uridine (NEG). Finally, among the differentially annotated metabolites in LHAJ and HPAJ, the most significantly up-regulated substance in LHAJ compared to HPAJ is (2R,3R)-3,5,7-trihydroxy-2-(3-hydroxyphenyl)-2,3-dihydrochromen-4-one, while the most significantly down-regulated substance was L-proline. The substance with the largest downregulation difference (blue point on the far left) is melissoidesin I. In the NEG, the substance with the most significant downregulation difference is 5-chlorosalicylic acid, which inhibits the renal tubular organic acid transporter, leading to interstitial nephritis.

#### 3.5.4 Metabolic pathway enrichment analysis

For metabolites with identification results, the number of metabolites was counted based on the major categories of KEGG metabolic pathways they participate in. Among the first-level classifications of KEGG pathways, the metabolic pathways had the most annotated metabolites, followed by genetic information processing and environmental information processing.

**Figure 7:**
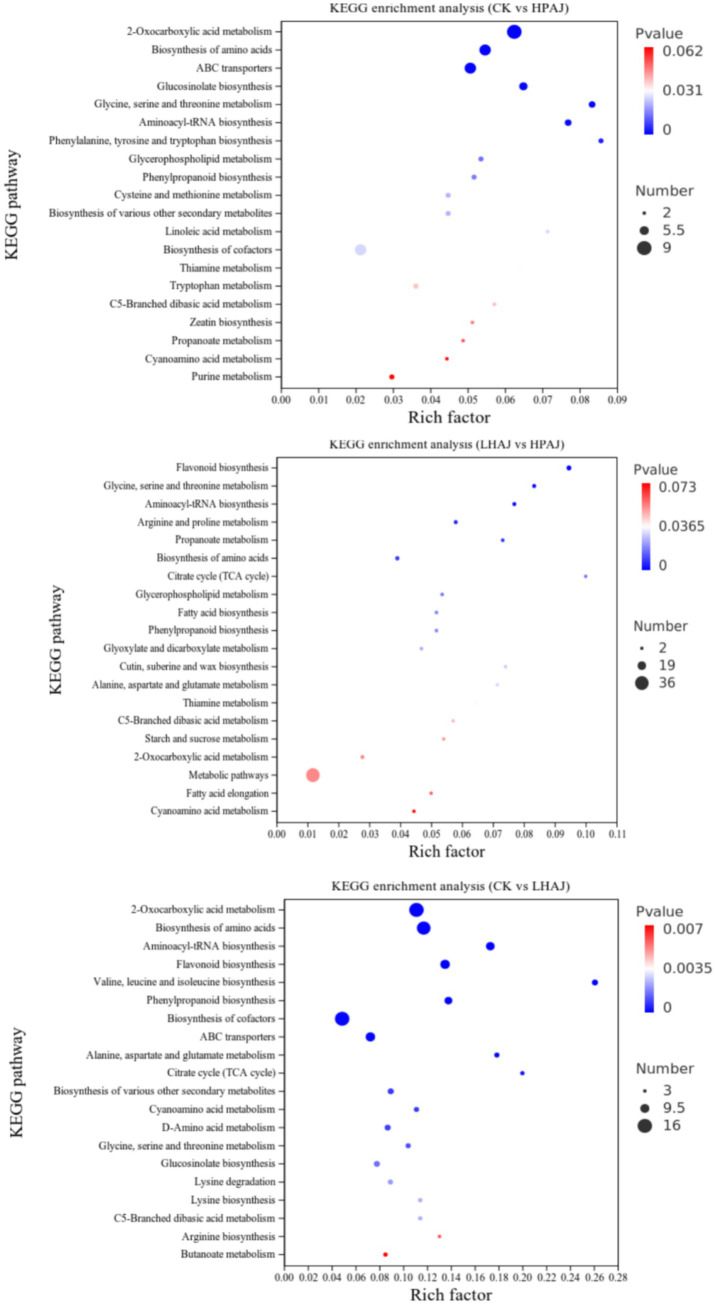
KEGG enrichment analysis diagram of CK and LHAJ, CK and HPAJ, LHAJ, and HPAJ. *n* = 3. *L. helveticus* R0052 fermented AJ (LHAJ); *L. helveticus* R0052 and *L. plantarum* fermented AJ as 1:2 (HPAJ).

**Figure 8:**
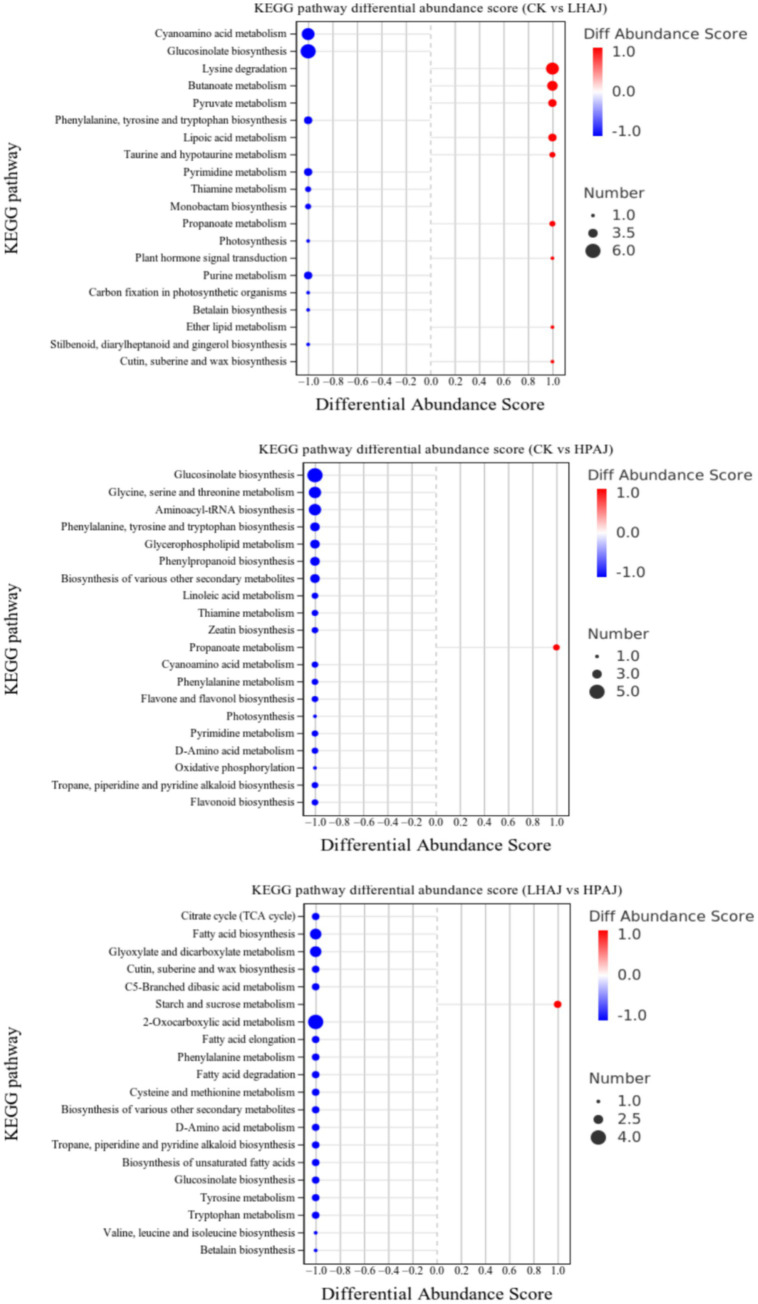
KEGG pathway differential abundance scores of CK and LHAJ, CK and HPAJ, LHAJ and HPAJ. *n* = 3. *L. helveticus* R0052 fermented AJ (LHAJ). *L. helveticus* R0052 and *L. plantarum* fermented AJ as 1:2 (HPAJ).

Pathway enrichment analysis of HPAJ metabolites was performed using the KEGG database, with apple (*Malus domestica*) as the target species. Using the KEGG database, the top 20 pathways with the highest enrichment significance were screened (Figure 7). In the CK vs. LHAJ comparison, the most significantly enriched pathways were valine, leucine, and isoleucine biosynthesis; the TCA cycle; and alanine, aspartate, and glutamate metabolism. In the CK vs. HPAJ comparison, the most significantly enriched pathways were phenylalanine, tyrosine, and tryptophan biosynthesis, glycine, serine, and threonine metabolism, and aminoacyl-tRNA synthesis. Finally, when comparing HPAJ with LHAJ, the most significantly enriched pathways were the TCA cycle, glycine, serine, and threonine metabolism, and flavonoid biosynthesis. Further analysis of the regulation of metabolites in enriched metabolic pathways was conducted by calculating the differential abundance scores for each metabolic pathway, as shown in Figure 8. In all three comparison scenarios, the expression trends of all identified metabolites in each pathway were either up-regulated or down-regulated. Compared with LHAJ, the metabolic pathway with the most up-regulated metabolites in CK was lysine degradation, while the metabolic pathway with the most down-regulated metabolites was the biosynthesis of glucosinolates. Compared with HPAJ, most pathways were downregulated in CK, with the most upregulated pathway being pyruvate metabolism and the most downregulated pathway being the biosynthesis of glucosinolates. Compared with HPAJ, the most upregulated pathway in LHAJ was starch and sucrose metabolism, and the most downregulated pathway was 2-oxoglutarate metabolism.

The TCA cycle is the central hub of material metabolism in living organisms (40). This pathway involves the breakdown of glucose or other fermentable sugars into pyruvate through glycolysis. Under aerobic conditions, pyruvate is further oxidized to produce energy, while under anaerobic fermentation conditions, pyruvate is catalyzed by lactate dehydrogenase to convert into lactate. Some metabolites in the alanine, aspartate, and glutamate metabolic pathways, such as aspartate, D-glucose-6-phosphate, and succinate, are also present in the TCA cycle and the nicotinic acid and nicotinamide metabolic pathways. Additionally, L-aspartic acid is a core metabolic product in the *β*-alanine and histidine metabolic pathways, playing a role in maintaining metabolic balance and growth rate in LAB (41). Under single fermentation conditions, a significant upregulation of succinic acid was observed in LHAJ, and LHAJ also exhibited higher acidity values than other samples in electronic tongue analysis. In the biosynthesis pathways of phenylalanine, tyrosine, and tryptophan, flavonoid compounds are synthesized from phenylalanine, which then enters the flavonoid-anthocyanin glycoside pathway (42). The biosynthesis of valine, leucine, and isoleucine, as well as the metabolism of alanine, aspartic acid, and glutamic acid, indicate that during fermentation, probiotics prioritize the utilization of free amino acids as a nitrogen source, promoting their own proliferation and metabolic activities, leading to rapid consumption of amino acids and a significant decrease in free amino acid content. By the mid-to-late stages of fermentation, amino acid levels stabilize, and microbial metabolism may break down proteins to release new amino acids (43).

Compared to CK, the biosynthesis of glucosinolates is the same pathway among the most downregulated metabolites in LHAJ and HPAJ. The biosynthesis of glucosinolates involves multiple enzymatic reactions, including amino acid precursor modification, glycosylation, and sulfation. Compared to HPAJ, LHAJ has more metabolites in the starch and sucrose metabolic pathways. 2-Oxocarboxylic acids (*α*-keto acids) are a class of compounds that play a central hub role in biological metabolism. 2-Oxocarboxylic acid metabolism connects carbohydrate, lipid, and amino acid breakdown with oxidative phosphorylation in the TCA cycle. Pyruvate can be oxidized aerobically to produce acetyl-CoA, which enters the TCA cycle, and α-ketoglutarate dehydrogenase generates succinyl-CoA. Alanine and glucose can produce pyruvate through transamination or gluconeogenesis. Glutamic acid and proline can produce α-ketoglutarate through specific reactions.

In summary, compared to the unfermented control (CK), both HPAJ and LHAJ induced distinct metabolic reprogramming. In this study, LHAJ uniquely enriched pathways for amino acid biosynthesis (e.g., phenylalanine) and specialized secondary metabolism (e.g., glucosinolate biosynthesis), suggesting a divergent strategy oriented towards flavor and functional compound synthesis from the outset. This is also attributable to the fact that *L. helveticus* is an amino acid-deficient strain requiring 14 essential amino acids. It possesses a highly efficient proteolytic system, relying on this system to obtain all amino acids (AAs) necessary for its growth (44). In contrast, HPAJ broadly activated glycolysis and oxidative phosphorylation, priming it for intensive acid production. The critical insight emerged from the direct HPAJ vs. LHAJ comparison, which pinpointed the TCA cycle as the key discriminant—being highly active only in HPAJ. This indicates that HPAJ’s fermentation engages a complete oxidative metabolism for efficient and diverse acid generation, whereas LHAJ appears to utilize a more limited acid-producing route centered on pyruvate metabolism, instead channeling resources into amino acid transformation. Consequently, HPAJ’s lower pH and sharper acidity are direct outcomes of vigorous oxidative acid synthesis (Figure 2), while LHAJ’s potentially more complex flavor profile may originate from its active amino acid and specialized metabolic pathways. This demonstrates that the choice of fermentation microbiota can steer apple juice metabolism down decisively different pathways, leading to products with targeted sensory and functional properties.

## 4 Discussion

KEGG analysis uncovered a critical metabolic bifurcation. HPAJ was characterized by a significant upregulation of the TCA cycle and related energy metabolism, indicating a robust oxidative metabolism geared towards efficient acid production. This aligns with its lower pH and higher titratable acidity. In stark contrast, LHAJ showed preferential enrichment in biosynthesis pathways of amino acids (e.g., valine, leucine, isoleucine, phenylalanine) and specialized plant secondary metabolites. This partition suggests that while HPAJ channels carbon flux towards central energy and acid generation, LHAJ allocates more resources to the synthesis of amino acids (45), which serve as direct precursors for a wide array of flavor-active compounds (46). The activated TCA cycle in HPAJ supplies ample acetyl-CoA and other organic acid precursors. This likely fuels the synthesis of medium- and long-chain ethyl esters (e.g., ethyl caprylate, ethyl caprate, phenethyl acetate) and branched-chain esters (e.g., isoamyl acetate) found uniquely or in much higher concentrations in HPAJ (Table 1; Figure 3). This observation aligns with prior studies on the fermentation of apple juice using *L. plantarum* (35, 47). The concurrent high levels of phenylacetaldehyde (rose/honey odor) further suggest an efficient Ehrlich pathway degradation of phenylalanine, a pathway initiated from the enriched aromatic amino acid biosynthesis. Consistent with its enriched amino acid biosynthesis, LHAJ exhibited higher or unique levels of compounds directly derived from amino acid metabolism. This includes higher concentrations of 1-hexanol and certain branched-chain esters (e.g., ethyl 2-methylbutyrate), which can originate from leucine and isoleucine metabolism. 3-Methyl-1-butanol and 2-methyl-1-butanol are derived from leucine and isoleucine, respectively, and are formed by the reduction of their corresponding aldehyde (49). The significant presence of 3-methyl-1-butanol and 2-methyl-1-butanol further supports active branched-chain amino acid catabolism.

## 5 Conclusion

In this study, the effects of single- and mixed fermentation on the sensory and volatile components of AJ were investigated, and non-targeted metabolomics technology was used to explore the main differential metabolites in AJ fermented under different conditions and the metabolic pathways reflected by these metabolites. Mixed fermentation was superior to single fermentation in terms of colony count, sensory score, and flavor. Analysis of volatile compounds revealed that mixed fermentation significantly enhanced the aroma and flavor profile of the juice product. LAB fermentation markedly improved the overall quality of AJ, intensifying fruity, sweet, and herbal notes to create a more pleasant overall aroma. The mixed fermentation promoted the production of common aroma substances and contributed to the production of esters and acids. The flavor disparity between LHAJ and HPAJ is not stochastic but is programmed by distinct underlying metabolic logic. HPAJ’s fermentation, driven by a more complete oxidative metabolism (TCA cycle), is optimized for the high-yield production of complex esters and aromatic aldehydes, resulting in a strong, mature, and florally complex aroma profile. Conversely, LHAJ’s metabolism, emphasizing amino acid anabolism and specialized pathways, leads to a profile richer in varied alcohols and simpler fruit esters, yielding a different but potentially more delicate aromatic complexity.

In conclusion, this study demonstrates that lactic acid fermentation effectively enhances the sensory profile and beneficially alters the composition of apple juice. Future research should focus on elucidating the specific beneficial metabolites generated via distinct metabolic pathways during fermentation. By linking these metabolites to targeted health outcomes, the development of precisely designed functional beverages can be advanced. However, the practical realization of these functional claims requires further systematic investigation.

## Data Availability

The original contributions presented in the study are included in the article/Supplementary material, further inquiries can be directed to the corresponding author.

## Footnotes

1
https://www.mzcloud.org

2
https://www.lipidmaps.org

3
https://hmdb.ca

4
https://mona.fiehnlab.ucdavis.edu

5
https://www.genescloud.cn
