# Supplementary material for: Mixed fermentation improved apple juice quality: juice characteristics and mechanism
Source: Front Nutr. 2026 Jan 27;13:1730713. doi: 10.3389/fnut.2026.1730713 (PMC12890255; doi:10.3389/fnut.2026.1730713)
Supplement: Supplementary file 1 [file Table_1.docx]

***Supplementary materials***

| Table S1. Instruments and equipment | | |
| --- | --- | --- |
| Name | Model | Manufacturer |
| Fruit washer | DHXG-3 | Shangdong Dehui Fermentation Intelligent Equipment Co., LTD, Shandong, China |
| Crusher | DHFS-1 | Shangdong Dehui Fermentation Intelligent Equipment Co., LTD, Shandong, China |
| Extractor | DHZZ-1 | Shangdong Dehui Fermentation Intelligent Equipment Co., LTD, Shandong, China |
| Agricultural products storage and freezer | BF8 | BITZER Refrigeration Technology (Shanghai) Co., Shanghai, China |
| Chromatographic column | ECOSIL NH2 120A5μm 250X4.6mm NH1205-2546 | Guangzhou Lubex Scientific Instrument Co., LTD, Guangzhou, China |
| Water bath | HH-600 | Shanghai Haozhuang Instrument Co., LTD, Shanghai, China |
| Handheld Refractometer | PAL-1 | Atago Co., Tokyo, Japan |
| Headspace-solid phase microextraction gas chromatography-mass spectrometry | Agilent 7890B-7000D | Shimadzu Corporation, Tokyo, Japan |
| Freeze Centrifuge | Eppendorf 5430R | Eppendorf |
| Ultra-High Performance Liquid Chromatograph | Vanquish Flex | Thermo Fisher Scientific Inc. |
| High-Resolution Mass Spectrometer | Orbitrap Exploris 120 | Thermo Fisher Scientific Inc. |
| High-Throughput Tissue Grinder | TL-48R | Shanghai Jingxin Industrial Development Co., Ltd. Shanghai, China |

| Table S2. Scoring rules for sensory evaluation of fermented apple juice | | |
| --- | --- | --- |
| Sensory indicators | Scoring standard | Score |
| Organisational status (15 points) | Juice is homogeneous, no suspension or sedimentation | 12-15 |
|  | Juice with a small amount of suspension or precipitation | 8-11 |
|  | Juice is visibly layered with a small amount of sediment present | 4-7 |
|  | Juice is clearly layered with a large amount of sediment present | 0-3 |
| Colour (15 points) | Uniform, bright yellow colour | 12-15 |
|  | Uniform colour, light yellow, dark yellow or brown | 8-11 |
|  | Uneven colour, bright yellow | 4-7 |
|  | Uneven colour, light yellow, dark yellow or brown. | 0-3 |
| Flavour (20 points) | Strong aroma, with prominent apple fruit flavour. | 16-20 |
|  | Light aroma, with a slight apple fruit flavour | 11-15 |
|  | Faint aroma, almost no apple fruit flavour. | 6-10 |
|  | Obvious off-flavour | 0-5 |
| Sweet and sour suitability (20 points) | Juice is moderately sweet and sour | 16-20 |
|  | Juice is slightly sour or sweet | 11-15 |
|  | Juice is too acidic or too sweet | 6-10 |
|  | Juice is too acidic or too sweet | 0-5 |
| Taste (30 points) | Juice has a prominent apple flavour and no off-flavour | 22-30 |
|  | Juice has a more pronounced apple flavour and no off-flavour | 16-22 |
|  | Juice has little apple flavour and no off-flavour | 8-15 |
|  | Juice has no apple flavour and a pronounced off-flavour | 0-7 |

| Table S3. Positive and negative mode elution gradients | |
| --- | --- |
| Time (min) | B % |
| 0 | 5% |
| 1 | 5% |
| 4.7 | 95% |
| 6 | 95% |
| 6.1 | 5% |
| 8.5 | 5% |

| Table S4. C-PEN3 Electronic nose sensors and response components | | |
| --- | --- | --- |
| Array number | Sensor name | Response component |
| 1 | W1C | Aromatic components, benzene |
| 2 | W5S | High sensitivity, sensitive to nitrogen oxides |
| 3 | W3C | Aromatic components sensitive, amines |
| 4 | W6S | Selective mainly for hydrides |
| 5 | W5C | Aromatic component of short-chain alkanes |
| 6 | W1S | Sensitive to methyl groups |
| 7 | W1W | Sensitive to sulphides |
| 8 | W2S | Sensitive to alcohols, aldehydes and ketones |
| 9 | W2W | Aromatic component, sensitive to organosulfides |
| 10 | W3C | Sensitive to long chain alkanes |

| Table S5. Sensor-to-taste value mapping information | | | |
| --- | --- | --- | --- |
| Sensor | Corresponding taste | Taste information | |
|  |  | First Taste | Aftertaste |
| C00 | Acidic bitterness | Bitterness | Aftertaste-Bitterness |
| AE1 | Astringency | Astringency | Aftertaste-Astringency |
| CA0 | Sourness | Sourness | × |
| CT0 | Saltness | Saltness | × |
| AAE | Umami | Umami | Richness |

| Table 1. Aromatic volatile compounds in CK, LHAJ and HPAJ. Results are expressed as mean ±standard deviation, n=3. ND, not detected. Different letters in the same row signify significant differences (p <0.05). L. helveticus R0052 fermented AJ (LHAJ); L. helveticus R0052 and L. plantarum fermented AJ as 1:2 (HPAJ). | | | | | | |
| --- | --- | --- | --- | --- | --- | --- |
| Number | | Volatile compounds | Retention time (min) | Concentrations (μg·L^-1^) | | |
|  |  |  |  | CK | LHAJ | HPAJ |
|  | | Esters | | | | |
| 1 | | Ethyl acetate | 7.15 | 112±14^c^ | 169±7^b^ | 1,030±23^a^ |
| 2 | | Formic acid, 2-propen-1-yl ester | 7.76 | ND | ND | 1728±145 |
| 3 | | Ethyl propionate | 8.50 | 163±17^a^ | 189±40^a^ | 156±11^a^ |
| 4 | | Propyl acetate | 8.89 | 200±18^b^ | 276±40^a^ | ND |
| 5 | | Methyl 2-methylbutyrate | 9.84 | 47.8±6.8^b^ | 58.6±5.2^a^ | ND |
| 6 | | Isobutyl acetate | 9.85 | 33.6±5.0^b^ | 38.4±6.4^b^ | 207±30^a^ |
| 7 | | Ethyl butyrate | 10.65 | 775±63^a^ | 901±98^a^ | 344±53^b^ |
| 8 | | Ethyl isobutyrate | 10.68 | 133±0^a^ | ND | 74.03±0.02^b^ |
| 9 | | Propyl propionate | 10.79 | 100±6^b^ | 163±3^a^ | 28.1±0.9^c^ |
| 10 | | Ethyl 2-methylbutyrate | 11.10 | 1850±151^a^ | 2070±238^a^ | 526±114^b^ |
| 11 | | Butyl acetate | 11.87 | 1180±145^a^ | 1240±360^a^ | ND |
| 12 | | 2-Methylbutyl acetate | 13.40 | 3350±233^b^ | 3990±237^a^ | ND |
| 13 | | Isoamyl acetate | 13.42 | ND | ND | 6810±1010 |
| 14 | | Propyl butyrate | 13.45 | 109±4 | ND | ND |
| 15 | | Ethyl valerate | 13.69 | 123±13^a^ | 37±2^b^ | 42.6±4.6^b^ |
| 16 | | Butanoic acid,2-methyl-, propyl ester | 14.02 | 453±25^b^ | 632±2^a^ | 195±38^c^ |
| 17 | | Aceticacid | 15.08 | 75.1±15.3^a^ | 93±10^a^ | 28.9±9.3^b^ |
| 18 | | Butyl butyrate | 16.77 | 80.8±1.6^a^ | 72.1±12.8^a^ | ND |
| 19 | | 2-Methyl-propanoic acid propyl ester | 16.82 | 260±25^b^ | 408±34^a^ | ND |
| 20 | | Ethyl caproate | 17.48 | 6430±2750^a^ | 1950±13^b^ | 7540±284^a^ |
| 21 | | Hexyl acetate | 18.90 | 580±7.76^b^ | 317±39^b^ | 3190±400^a^ |
| 22 | | Propyl hexanoate | 20.62 | ND | 49.3±0.4 | ND |
| 23 | | Nitrous acid | 20.64 | 834±42 | ND | ND |
| 24 | | Formic acid | 20.72 | ND | ND | 3700±80 |
| 25 | | Ethyl heptanoate | 21.21 | 113±57^b^ | ND | 196±18^a^ |
| 26 | | Ethyl caprylate | 25.41 | ND | ND | 17300±1210 |
| 27 | | Ethyl caprate | 36.69 | ND | ND | 632±95.1 |
| 28 | | Ethyl 9-Decenoate | 38.84 | ND | ND | 258±51 |
| 29 | | Phenethyl acetate | 43.01 | ND | ND | 8050±147 |
| 30 | | 3-Hydroxy-2,2,4-trimethylpentyl isobutyrate | 44.27 | ND | ND | 32.1±2.1 |
|  | | Alcohol | | | | |
| 31 | | 3-Aminopropanol | 10.79 | ND | 162±1 | ND |
| 32 | | 2-Methyl-1-propanol | 12.17 | ND | ND | 337±24.7 |
| 33 | | 1-Butanol | 13.88 | ND | 737±22^a^ | 552±2^b^ |
| 34 | | 2-Methyl-1-butanol | 16.16 | ND | 910±265 | ND |
| 35 | | 3-Methyl-1-butanol | 17.76 | ND | 51.9±15.3 | ND |
| 36 | | 1-Hexanol | 21.72 | ND | 4310±130^b^ | 5290±208^a^ |
| 37 | | Cyclohexanol | 23.85 | 410±24^b^ | 651±10^a^ | ND |
| 38 | | 2-Hexen-1-ol | 23.85 | ND | 591±49 | ND |
| 39 | | 1,3-Dioxolane-2,2-diethanol | 24.37 | 65.6±8.4^a^ | 38.7±0.0^b^ | ND |
| 40 | | 1-Heptanol | 26.02 | ND | 31.9±0.2^b^ | 188±25^a^ |
| 41 | | 4-Methyl-1-hexanol | 26.03 | ND | ND | 168±17 |
| 42 | | 2-Propyl-1-pentanol | 27.81 | ND | ND | 431±32 |
| 43 | | 2-Ethylhexanol | 27.82 | 359±18^b^ | 214±75^c^ | 676±24^a^ |
|  | | Ketones | | | | |
| 44 | | 3-Hydroxy-2-butanone | 17.44 | ND | 1880±98 | ND |
| 45 | | 2-Hydroxycyclopent-2-en-1-one | 19.93 | 3730±90^a^ | 3930±651^a^ | 3690±366^a^ |
| 46 | | 6-Methyl-5-hepten-2-one | 21.42 | ND | ND | 154±38 |
| 47 | | 2-Imidazolidone | 36.60 | 55.2±3.1 | ND | ND |
| 48 | | beta-Damascenone | 43.17 | 223±3^c^ | 386±0^a^ | 256±16^b^ |
| 49 | | Damascenone | 43.18 | 205±23^b^ | 218±9^b^ | 260±10^a^ |
|  | Aldehydes | | | | | |
| 50 | | Hexanal | 11.99 | 471±93 | ND | ND |
| 51 | | 4-Methyl-3-pentene-1-one | 16.97 | 2160±590^a^ | 124±2^b^ | ND |
| 52 | | Benzaldehyde | 30.61 | 76.6±7.5^b^ | ND | 479±37^a^ |
| 53 | | Phenylacetaldehyde | 37.15 | ND | ND | 9950±1020 |
|  | | Acids | | | | |
| 54 | | Sarcosine | 13.30 | ND | 184±4 | ND |
| 55 | | 2-Methylhexanoic acid | 38.20 | 198±3^c^ | 203±3^b^ | 952±14^a^ |
| 56 | | 2-Methyl butyric acid | 38.21 | ND | 200±40^b^ | 607±126^a^ |
| 57 | | Valeric acid | 43.76 | ND | ND | 610±47 |
| 58 | | Butyric acid | 43.77 | ND | ND | 368±30 |
| 59 | | Octanoic acid | 48.48 | ND | ND | 971±145 |
|  | | Others | | | | |
| 60 | | 3-Ethoxy-1-propene | 7.76 | ND | ND | 279±24 |
| 61 | | 1,2-Bis(Difluoroamino)-2-Methylpropane | 11.76 | 362±386 | ND | ND |
| 62 | | 2-Amino-2-cyanoacetamide | 13.86 | 649±72 | ND | ND |
| 63 | | Diazoethane | 13.90 | ND | 1026±45 | ND |
| 64 | | 2-Methyl-2-nitropropane | 16.34 | ND | ND | 9980±1047 |
| 65 | | Ethoxyethyne | 16.38 | ND | 87.4±4.7^b^ | 31100±7970^a^ |
| 66 | | 1-Isopropyl-2-methylbenzene | 25.80 | ND | ND | 454±16 |
| 67 | | 1,2,3,5-Tetramethylbenzene | 25.84 | ND | 95.5±2.8^b^ | 425±57^a^ |
| 68 | | 2-Acetylfuran | 26.37 | 28.2±1.0 | ND | ND |


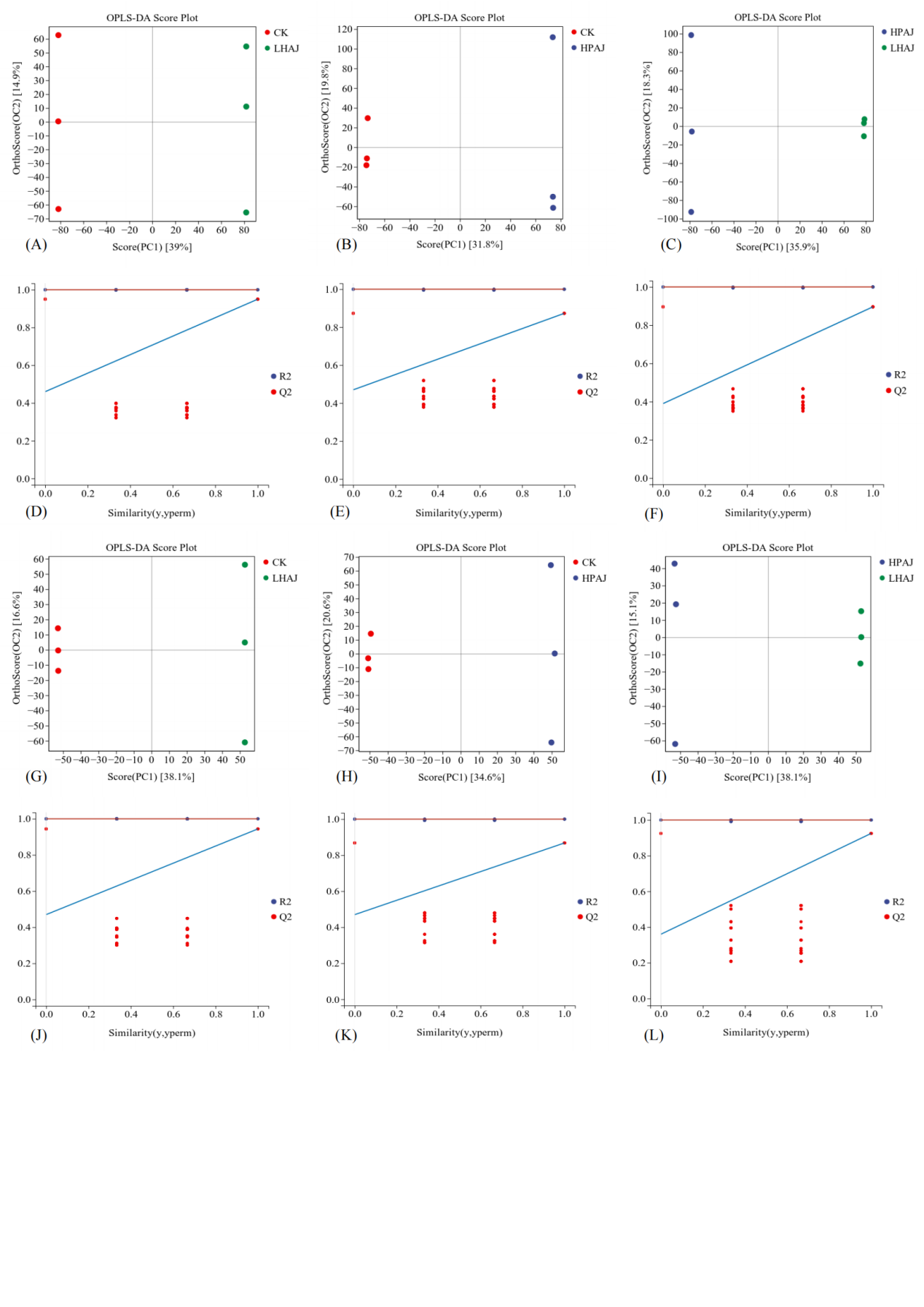


Figure S1. Multivariate statistical analysis of metabolite differences of CK and LHAJ, CK and HPAJ, LHAJ and HPAJ. Optimized projection linear discriminant analysis (OPLS-DA) score plot (POS: A, B, C. NEG: G, H, I). Permutation test results (POS: D, E, F. NEG: J, K, L). *L. helveticus* R0052 fermented AJ (LHAJ); *L. helveticus* R0052 and *L. plantarum* fermented AJ as 1:2 (HPAJ).


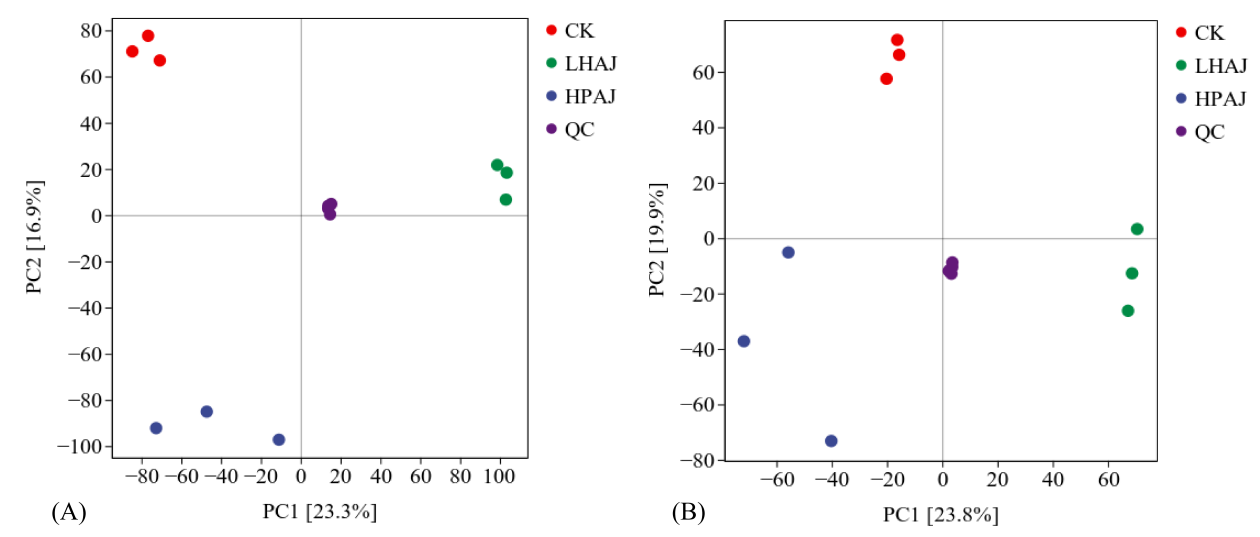


Figure S2. Overall PCA analysis (POS:A, NEG:B). Control check (CK). *L. helveticus* R0052 fermented AJ (LHAJ); *L. helveticus* R0052 and *L. plantarum* fermented AJ as 1:2 (HPAJ).
